# Reply to Comment on “Fast and accurate electromagnetic field calculation for substrate-supported metasurfaces using the discrete dipole approximation”

**DOI:** 10.1515/nanoph-2024-0302

**Published:** 2024-06-14

**Authors:** Weilin Liu, Euan McLeod

**Affiliations:** Wyant College of Optical Sciences, 12330 University of Arizona, 1630 E University Blvd, Tucson, AZ 85719, USA

**Keywords:** discrete dipole approximation, Sommerfeld integral, cylindrical Green’s function

## Abstract

The recent comment on our previously published article questioned the novelty and computational efficiency of our work. Here we respond by restating the novelty and scientific value of our work as well as showing why the specific alternative methods stated in the comment are unlikely to outperform the methods we compare for metasurface applications involving high refractive index particles near high refractive index substrates.

We appreciate the interest taken in our article by Dr. Chaumet, and here respond to his comment [1] on our recently published manuscript [2].

We begin by noting that in our original article, we did not claim the discovery of a fundamentally novel method, but instead, that the value of our publication is in the speed-performance tradeoff comparison of several methods, in particular for the case of high refractive index particles and/or high refractive index substrates. We are also well aware of the large body of work in the last few decades on the discrete dipole approximation (DDA), and cited 48 references in our paper, including some work by Dr. Chaumet and some of the references cited in his comment. The scientific value of our paper was clearly stated in the second-to-last paragraph of our introduction [2]:

…we compare the accuracy and computation speed between two-dimensional (2D) and one-dimensional (1D) methods of computing the Sommerfeld integrals for multiple dipoles. Our approach takes advantage of parallel processing for faster calculations and outputs both the transmitted and reflected fields, which, to the best of our knowledge, is not done in other existing simulation tools. We also evaluate the speed and accuracy of four different polarizability models: the simple Clausius–Mossotti relation, the radiation reaction correction, the lattice dispersion relation, and the digitized Green’s function. Using our 1D integration method and the radiation reaction correction dipole model, we find highly accurate and rapid near-field and scattered field calculation for light incident on elliptical cylinder (“pillbox”) shaped nanoparticles that are sitting on a substrate. This holds true for metallic and high-index dielectric particles and/or substrates.

For the methods we compare, although the underlying algorithms are not novel, the implementation is our own (except for FDTD), and created from scratch. This ensured fair comparisons among methods, and there are several technical details related to our implementation that differ from previous works, as noted by Dr. Chaumet. However, as we elaborate below, in contrast to Dr. Chaumet’s comment, our implementation of these technical details is not inferior to previous implementations.

In his comment, Dr. Chaumet suggests that, when calculating the Green’s function for a dipole in close proximity to a substrate, the poles in the Sommerfeld integral can be removed by a change of variables. He cites that this change of variables has been performed in [3], [4], [5]. However, closer inspection reveals that there is no substrate considered in [3], and therefore no poles in even the initial problem. In [4], there is a substrate, but the change of variables is only used to calculate the total radiated power, which is a simpler calculation than that of the full Green’s function. Ref. [5] does indeed suggest the use of the change of variables to remove the poles of the Green’s function in the presence of a substrate, but no derivation is provided in that paper, only a reference to [6].

In [6], instead of integrating over radial spatial frequencies, *k*_*ρ*_, the integration variable is changed to:

(1) $$k_{0z}=\sqrt{k_{0}^{2}-k_{\rho }^{2}},$$

where *k*_0_ is the wavenumber in medium 0. In addition to this change of variables, the path of integration is split into two paths:

(2) $$G_{i}=\left(-\overset{\sqrt{\varepsilon }k_{0}}{\underset{0}{\int }}+\overset{i\infty }{\underset{0}{\int }}\right)f_{4}\left(k_{0z}\right)dk_{0z}.$$

The intersection of these two paths at *k*_0*z*_ = 0 corresponds to one of the poles of the original integrand, which occurred at *k*_*ρ*_ = *k*_0_. Placing poles at the boundaries between integration intervals is something that we do in our manuscript too, using the Gauss–Kronrod (GK) method, but with a more straightforward, direct integration path along the real axis for the radial spatial frequency. We also note that even after the change of variables, poles potentially remain within the integrand. For example, in Eq. (24) of [6], the denominator of the integrand is $\Delta_{p1}\Delta_{p2}-e^{-2ik_{0z}d}$ , where Δ_*p*1_ and Δ_*p*2_ are Fresnel reflection coefficients and *d* is a layer thickness. For some parameter combinations, this denominator can equal zero, resulting in poles in the integrand.

Dr. Chaumet mentions that there is an even more efficient way to compute the Green’s tensor, which was presented by Paulus, Gay-Balmaz, and Martin [7]. As with the most efficient method in our paper, Paulus’s approach begins by writing the Sommerfeld integral in cylindrical coordinates, where the challenging integral runs from *k*_*ρ*_ = 0 to *k*_*ρ*_ = ∞. As stated in Eq. (17) of Ref. [7], a typical integrand has the form:

(3) $$\begin{array}{ll} G\left(k_{\rho };r,r^{\prime }\right) & =g\left(k_{\rho };r,r^{\prime }\right)J_{n}\left(k_{\rho }\rho \right)\left[A\left(k_{\rho },z^{\prime }\right)\exp\left(ik_{lz}z\right)\right.\\ & \;\left.+B\left(k_{\rho },z^{\prime }\right)\exp\left(-ik_{lz}z\right)\right], \end{array}$$

where $k_{lz}=\sqrt{k_{l}^{2}-k_{\rho }^{2}}$ and *k*_*l*_ is the wavenumber of medium *l*. Then, in contrast to our approach, [7] uses the residue theorem to select a new integration contour to avoid the poles and reduce oscillation in the Sommerfeld integrand. An elliptical contour in the fourth quadrant of the complex plane is used to avoid the poles, while the contour from the end of the elliptical part to ∞ is evaluated parallel to the imaginary axis. The elliptical contour is a clever idea, which provides the benefit of avoiding poles at the cost of added complexity in the geometrical definition of the contour. In our paper, we show that the GK approach, placing interval boundaries at the pole locations, is also a sufficient approach. Our approach is simpler in that no decisions are required about the size of the elliptical contour. A systematic comparison of the speed-accuracy tradeoff between the two approaches would be an interesting idea for a future study; it is currently unclear to us which would perform better.

The rationale for making the second part of the contour, the path from the end of the ellipse to complex ∞, parallel to the imaginary axis was that Bessel and Hankel functions do not exhibit oscillations when evaluated parallel to the imaginary axis. Although integrating parallel to the imaginary axis removes the oscillations from the Bessel and Hankel functions, it introduces high-frequency oscillations into another part of the integrand: the terms $e^{\pm ik_{lz}z}$ in Eq. (3). For large real *k*_*ρ*_, *k*_*lz*_ is imaginary and these terms in Eq. (3) would decay evanescently. However, for integration along imaginary directions, these terms become oscillatory rather than evanescent. In other words, by switiching from integration along the real axis to integration in the imaginary direction, the oscillatory terms in the integrand have not been removed, but instead just switched from the Bessel (or Hankel) function term to the exponential term.

In Paulus’s article [7], the validation and speed-performance tradeoff data is limited: they only show one validation figure (Figure 6), where the test case involved 4 material layers, all with identical optical properties, which means that there are no reflections from interfaces, and it is difficult to draw conclusions on the performance of such an approach in more complex cases, such as the ones we analyze in our recent paper [2]. We found that the oscillatory behavior of the integrand is particularly difficult to handle when either the dipoles are very close to the substrate and/or the substrate has high refractive index, because the evanescent damping is weaker in those cases. On the other hand, Paulus’s test case of index-matched substrates with a dipole relatively far from an interface is a less stringent test than what we show in our paper, see, for example Figure 3c of [2].

In his comment, Chaumet recommended employing the Gauss–Kronrod–Patterson (GKP) method as opposed to our GK method because the nested quadrature rule of the GKP method supposedly increases convergence. We would like to note that in contradiction to Chaumet’s comment, Matlab’s quadgk implementation does indeed use a nested algorithm, which can be confirmed by looking at the Matlab source code for quadgk [8]. Perhaps Chaumet may have confused the definitions of Gaussian quadrature and Gauss–Kronrod quadrature, because the usual Gaussian quadrature would require recomputation of all abscissas at each iteration. Regrettably, we cannot delve into a more detailed comparison here because Chaumet writes that he published the GKP implementation in 2020, but included an incorrect reference to a different paper from 2017.

Dr. Chaumet also writes that he proposed interpolating a cylindrical Green’s tensor to Cartesian coordinates in 2020, but again cites a 2017 paper [9]. In our paper [2], we interpolate the Green’s tensor used for *field* calculations, rather than the Green’s tensor used for building up the dipole coupling matrix. Although there is nothing revolutionary about the concept of interpolating from one coordinate system to another, performing the interpolation only during the final field calculation permits dipoles to be placed at arbitrary locations that need not correspond to a Cartesian grid. In metamaterial applications, there can be a substantial number of (*z*, *z*′) pairs, where *z* is the elevation of the observation plane, and *z*′ is the elevation of a dipole. In cases with many such pairs, interpolation of the coupling matrix leads to computational inefficiency, particularly without any parallel computing techniques. In contrast, our implementation remains efficient for larger numbers of (*z*, *z*′) pairs. Furthermore, our electric field interpolation approaches are entirely independent of the discretization factor of the object, as many applications may require only a very sparse electric field calculation at the monitor plane.

The ability to place dipoles at arbitrary locations together with the challenge of appropriately handling poles are also the reasons we avoided using solutions based on fast Fourier transforms (FFTs). While FFT-based solutions can be computationally efficient in some cases, they require the dipoles to be placed on a regular rectangular grid. For future work by our lab and other researchers, we are particularly interested in sparse, irregular arrangements of nanoparticles, such as those fabricated via optical tweezers [10], [11], where particles are not necessarily placed on a regular grid. For such problems, it is important to have efficient solvers that do not rely on a rectangular grid.

Dr. Chaumet also compares the computational speed of the Paulus approach to the approaches we compare in our paper [2], without ensuring that the level of accuracy remains the same. Any numerical technique can be arbitrarily fast if one sacrifices accuracy. It is especially challenging to ensure accuracy in the evanescent near-field of the nanostructures, which is highly relevant for metasurface applications and something we examine in detail in our paper. The near-field of a metasurface is particularly challenging because there are many dipoles in near-contact with the substrate, making the evanescent damping in the Green’s tensor quite weak, as we mentioned above. This is also a reason why the example Chaumet gives of a 12 μm sphere sitting on a surface is not really a proper comparison; almost all of the dipoles in that sphere are very far away from the surface, leading to large evanescent damping of the Green’s tensor and an easier Sommerfeld integral that can be more accurately calculated in shorter time.

Finally, we wish to reiterate the novelty of our work: we evaluated the speed-accuracy tradeoff for several DDA approaches and showed that they can be effectively applied to near field calculations for low and high index nanoparticles on low and high index substrates. Notably, we demonstrate that DDA can achieve comparable accuracy to FDTD in significantly reduced computational time, particularly for sub-micron objects. The ability for the approaches we compare to handle irregular nanoparticle placement allows these approaches to be used for applications that may be unsuitable for the codes highlighted by Dr. Chaumet. We hope that the results we presented will be of interest and use to others involved in nanophotonics.
